# Designing, implementing and analysing a virtual trial

**DOI:** 10.1186/1745-6215-16-S2-O82

**Published:** 2015-11-16

**Authors:** Lauren J Scott, Barnaby C Reeves, Usha Chakravarthy, Chris A Rogers

**Affiliations:** 1 University of Bristol, Bristol, UK; 2 The Queen’s University of Belfast, Belfast, UK

## Background

Neovascular age-related macular degeneration (nAMD) is a common eye condition that can cause severe sight loss and blindness. Active disease is treated monthly until it becomes inactive, but regular monitoring by a hospital ophthalmologist is required as reactivation is common. The ECHoES trial was designed to assess whether, after appropriate training, community optometrists could make decisions about nAMD reactivation, to the same standard as hospital ophthalmologists.

## Methods

ECHoES was a non-inferiority virtual trial that utilised an existing repository of images and data collected during the IVAN trial to create 288 patient profiles (vignettes). In a balanced incomplete block design, 96 participants (48 ophthalmologists and 48 optometrists) each reviewed 42 randomly allocated vignettes in a pre-determined order. Each vignette was viewed by 7 ophthalmologists and 7 optometrists.

The primary outcome was correct classification of nAMD reactivation, compared to a reference standard. Data were analysed using mixed model logistic regression, with professional group and vignette-order fitted as fixed effects, and participant and vignette as random effects. The non-inferiority margin was set at 10% assuming ophthalmologists would correctly assess 95% of their vignettes (0.298 on the log-odds scale).

## Results

Optometrists and ophthalmologists correctly classified 1702/2016 (84.4%) and 1722/2016 (85.4%) vignettes respectively (odds ratio 0.91, 95% confidence interval 0.66 to 1.25, p=0.543).

## Conclusion

The ability of optometrists to make nAMD retreatment decisions is non-inferior to that of ophthalmologists. The ECHoES trial was designed in response to a rapid trials funding call and is an example of an efficient trial with a novel design.

